# The ex-illiterate brain: The critical period, cognitive reserve and HAROLD model

**DOI:** 10.1590/S1980-57642009DN30300008

**Published:** 2009

**Authors:** Maria Vania Silva Nunes, Alexandre Castro-Caldas, Dolores Del Rio, Fernado Maestú, Tomás Ortiz

**Affiliations:** 1 Neurological Clinical Research Unit, Faculdade de Medicina de Lisboa, Lisbon, Portugal.; 2 Portuguese Catholic University. Lisbon, Portugal.; 3 Centro de Magnetoencefalografía Dr. Pérez Modrego. Facultad de Medicina, Madrid, España.

**Keywords:** Illiteracy, magnetoencephalography, cognitive reserve, brain asymmetry, language, HAROLD model

## Abstract

The lifelong acquisition of cognitive skills shapes the biology of the brain. However, there are critical periods for the best use of the brain to process the acquired information.

**Objectives:**

To discuss the critical period of cognitive acquisition, the concept of cognitive reserve and the HAROLD (Hemispheric Asymmetry Reduction in Older adults) model.

**Methods:**

Seven women who learned how to read and to write after the age of 50 (ex-illiterates) and five women with 10 years of regular schooling (controls) were submitted to a language recognition test while brain activity was being recorded using magnetoencephalography. Spoken words were delivered binaurally via two plastic tubs terminating in ear inserts, and recordings were made with a whole head magnetometer consisting of 148 magnetometer coils.

**Results:**

Both groups performed similarly on the task of identifying target words. Analysis of the number of sources of activity in the left and right hemispheres revealed significant differences between the two groups, showing that ex-illiterate subjects exhibited less brain functional asymmetry during the language task.

**Conclusions:**

These results should be interpreted with caution because the groups were small. However, these findings reinforce the concept that poorly educated subjects tend to use the brain for information processing in a different way to subjects with a high educational level or who were schooled at the regular time. Finally, the recruiting of both hemispheres to tackle the language recognition test occurred to a greater degree in the ex-illiterate group where this can be interpreted as a sign of difficulty performing the task.

## Abstract (pt)

A aquisição ao longo da vida de competências cognitivas moldam a biologia do cérebro. No entanto, existem períodos críticos para o cérebro processar melhor as informações adquiridas.

**Objetivos:**

Discutir o período crítico de aquisição cognitiva, o conceito de reserva cognitiva e o modelo HAROLD (Redução da Assimetria Hemisférica em Idosos).

**Métodos:**

Sete mulheres que aprenderam a ler e escrever após a idade de 50 anos (ex-analfabetos) e cinco mulheres com 10 anos de escolaridade regular (controles) foram submetidas a um teste de reconhecimento de palavras enquanto a atividade cerebral estava sendo registrada mediante magnetoencefalografia. As palavras foram ouvidas com o emprego de dois tubos plásticos conectados a cada orelha e as gravações foram feitas com um magnetômetro de cabeça inteira com 148 bobinas de registro.

**Resultados:**

Ambos os grupos tiveram desempenho semelhante na identificação das palavras-alvo. Análise do número de fontes de atividade no hemisfério esquerdo e no hemisfério direito revelou diferenças significativas entre os dois grupos, mostrando que os ex-analfabetos tiveram menor assimetria cerebral funcional no desempenho da tarefa.

**Conclusões:**

Estes resultados devem ser interpretados com cautela, pois os grupos são pequenos. No entanto, reforçam o conceito que os indivíduos com baixa escolaridade tendem a usar o cérebro para processar informações de uma forma diferente do que indivíduos com nível educacional elevado ou que tenham o adquirido na época regular. Finalmente, o recrutamento de ambos os hemisférios para reconhecer as palavras ocorreu mais intensamente no grupo de ex-analfabetos e pode ser interpretado como um sinal de maior dificuldade na tarefa.

It is now widely accepted that there are critical periods in life for the acquisition of different cognitive skills. This concerns both the acquisition of behavioral skills and the necessary biologic brain adaptation (for general reference, see Knudsen, 2004). We can better understand this process by studying individual cases or atypical populations. Cases such as Genie are classic demonstrations of the poor quality of these skills when acquired later than usual (Curtis, 1977). Adult illiterate subjects are another valuable source of information. In previous studies this group was shown to have poor development of the corpus callosum (Castro-Caldas et al., 1999) and also demonstrated different patterns of brain activation compared to non-illiterate adults while performing language tasks (Castro-Caldas et al. 1998). There is also evidence of a critical period for processing American Sign Language (Newman et al., 2001). We can therefore consider that those subjects not exposed to the necessary information during these critical periods do not shape the brain in the same way as those who are.

Acquiring skills during the right periods and enriching the brain with information are the principles that underpin the concept of cognitive reserve. Stern (2002) defined this concept as “*the ability to optimize or maximize performance through differential recruitment of brain networks, which perhaps reflects the use of alternate cognitive strategies. Since the changes in brain recruitment associated with reserve are a normal response to increased task demands, this definition suggests that cognitive reserve is present in both healthy individuals and those with brain damage”* (p. 451).

The differential recruitment of brain networks is the basis of the HAROLD model proposed by Cabeza (2002) The HAROLD Model (Hemispheric Asymmetry Reduction in Older adults) states that old people will rely on bi-hemispheric processing in situations where unilateral processing is sufficient for young subjects (Cabeza et al., 2002). This model is best documented concerning the Pre Frontal Cortex and more developed in the domain of memory studies. Results of several other studies indicate that the reduction of hemispheric asymmetry associated with aging is a general feature of the aging brain (Nielson et al. 2002; Grady et al. 2000; Maguire and Frith, 2003), reflecting a change in the cognitive architecture.

The precise meaning of this change in processing is not clear. Using a behavioral paradigm, Reuter Lorenz et al. (1999), concluded that in the presence of a highly demanding task, subjects had an advantage if they recruited both hemispheres. Therefore, the recruitment of both hemispheres for solving a task that could be solved with one side of the brain in younger age groups can be envisaged as a mechanism which is compensatory in nature (for general discussion of this topic see Dasselar and Cabeza, 2005).

In our current research project we had the opportunity to study a rare population of subjects that decided to learn how to read and write after the age of 50, after being illiterate all their lives (they had never attended school for social reasons). They are excellent examples of individuals who have missed the critical period and because they are now older then 65 they can teach us how elderly subjects use their brain to deal with this newly acquired skill.

## Material and methods

### Subjects

Twelve neurologically intact Portuguese female adult volunteers, with no history of psychomotor developmental disabilities that could have prevented normal learning in childhood, were included in this study. Current or recent psychiatric or neurological illnesses were ruled out on a clinical basis. Before inclusion, and after the nature of the study had been fully explained to them, all participants signed consent forms which included agreement to travel by plane to Madrid, where the study was conducted.

The target population was composed of seven women selected from special programs for adult learning. They were unable to attend school at the usual age and had been completely illiterate throughout their lives. When they were freer from their family obligations (by the age of 50) they decided to go to school to learn how to read and write. It is important to note that the success rate on these programs is very low. Most of the participants drop out after the first few months because this constitutes a difficult process. However, some of the participants achieve a reasonable level of performance in reading words and small texts and writing simple messages. It was therefore very hard to find a large group suitable for the present study. The mean age of the study group was 70.86±7.4. The five controls had more than 10 years of schooling and attended school at the usual age (mean age: 73±9.6). This was also a difficult group to form because older educated women were not interested in participating in a research project in which they had to fly from Lisbon to Madrid and stay there for a couple of days while being submitted to examinations. We can therefore conclude that this was the possible group and that the results were sufficiently interesting to warrant being reporting. It is still important to note that, in these programs for adults, men are very rare and for cultural reasons avoid revealing their handicaps.

All participants were right handed according to the Humphrey Laterality questionnaire (modified by Hecaen & Ajuriguerra, 1963) and all scored above cut-off values for the Portuguese adaptation of the Mini Mental State (MMS) (Guerreiro et al., 1994). All were independent in daily life activities, and careful clinical assessment ruled out the diagnosis of dementia.

### MEG procedure

All the recordings took place at the Center of Magnetoencephalography Dr. Pérez Mondrego in Madrid, which is experienced in the language testing procedure (Maestú et al., 2002). Numerous studies have shown MEG to be a good method for studying mental function, particularly language. The capacity of MEG to determine hemispheric dominance has been tested through a series of studies in healthy controls (Breier et al., 1998, 2001; Simos et al., 1998) and patients. The results of MEG mapping were compared with either the results of the Intracarotid Amytal Procedure (IAP) (Breier et al., 2001; Maestú et al., 2002) or direct cortical stimulation mapping, performed either intraoperatively or extraoperatively through implanted electrode grids. In general, these studies showed that MEG is a valid tool for mapping the cerebral regions involved in mental functions.

### Stimuli and tasks

The procedures for MEG-language recordings have been described in detail elsewhere (Breier et al., 1998, 2001; Maestú et al., 2002), and will be briefly summarized here. For our purposes it suffices to emphasise that language specific brain activity was elicited using an auditory recognition task. This material was first devised in English then adapted to the Spanish language (Maestú et al., 2002). The present material was adapted from the Spanish version to Portuguese while the recording procedures were the same (Maestú et al., 2002). A list of 63 spoken abstract Portuguese nouns (real words) was stored on a Neuroscan STIM stimulation system (Neurosoft, Inc., El Paso, TX). The stimuli were arranged in three lists containing a total of 43 words: 33 “target” words that were the same in all three lists plus 10 distractors (also real words) that were unique in each list, giving a total of 129 auditory events. In order to avoid creating additional differences between the adapted material and the original, the same lexical items were retained in the adapted material. Concerning the frequency of the words, and regardless of any variability arising from their polysemic nature, all the words had high or at least medium frequency according to a *corpus* of the Portuguese language (ELAN Sub-corpus). Although no previous training was given, the task was explained in great detail to the participants. All the words were read aloud to the participants before testing to ensure they understood their meaning. The test was started on the scanner by presenting the 33 target words orally for coding immediately before the recording. The recording started simultaneously with the sequential presentation of the 3 sets of 43 words each. Subjects were asked to raise their right index finger whenever they recognized a target word (i.e. a target word presented before scanning and present in all three sets of words).

In this auditory task, spoken words were delivered binaurally via two 5-m- long plastic tubs terminating in ear inserts, at an 80-dB sound pressure level which was measured at the participants’ outer ear.

### MEG recordings and analysis

In recording sessions, participants were asked to lie motionless on a bed. Recordings were made in a magnetically shielded room with a whole head magnetometer (Magnes 2500; 4D Neuroimaging, San Diego, CA) consisting of 148 magnetometer coils placed in a cryogenic dewar container. The instrument is housed in a magnetically shielded room designed to reduce environmental noise.

The signal was filtered online with a band pass filter between 0.1 and 0.5 Hz digitized for 1000 ms (250 Hz sampling rate) including a 150 ms prestimulus period and subjected to an adaptative filtering procedure that is a part of the 4-D Neuroimaging package. These steps are necessary to minimize the amount of low frequency magnetic noise that is usually present in Magnetoencephalography recordings. The single trial event-related fields (ERFs) were averaged after removing those which occurred during an eye movement or blink (as indicated by peak to peak amplitude in the electrooculogram in excess of 50 μV). A minimum of 90 ERF epochs were used to calculate each waveform. The averaged waveforms were digitally filtered with a low pass 20 Hz filter. The intracranial sources of the observed ERFs, henceforth referred to as activity sources, were modeled as single equivalent current dipoles (ECDs), which were fitted at successive 4 msec intervals by using a nonlinear Levenberg-Marquardt algorithm. This algorithm was used for the ECD that most probably produced the observed magnetic field at any given point in time. The ECD computation was restricted to latency periods in which a single pair of magnetic flux dominated the left and/or the right half of the head surface. For any point in time the ECD fitting algorithm was applied to the magnetic flux obtained from a group of 34 to 38 magnetometers, always including both extremes.

The ECD solutions were considered satisfactory after meeting a correlation coefficient of at least 0.90 between the observed and the best predicted magnetic field distribution and a goodness fit of at least 0.9 or higher. The ECD locations were computed with reference to a Cartesian system defined by a set of three anatomical fiduciary points: the two external *meati* and the *nasion*.

In order to determine the anatomical regions where the activity sources were located, ECD coordinates were overlaid onto T1-weight, magnetic resonance (MR) im ages (TR 13.6 ms; TE 4.8 ms; recording matrix 256×256 pixels, 1 excitation, 240 mm field of view, and 1.4 mm slice thickness) obtained from every participant in a separate session. The MEG-MRI overlay was performed using the STAR program, which is part of the 4-D Neuroimaging software (see Maestú et al., 2002, for a detailed description of the co-registration process).

Early sources related to sensory processing (i.e., those that occur during the course of the M50 and M100 components), were analyzed separately to demonstrate the absence of auditory perceptual deficits in each subject. As it is believed that this early activity reflects initial sensory processing of the word stimuli (Breier et al., 1998) the number of activity sources that we considered in order to calculate activation in the left and right hemisphere were restricted to sources 150 ms or later following stimulus onset.

The Mann-Whitney test was used for comparisons of the number of sources between groups while the Wilcoxon test was used for intra-group comparisons.

## Results

### Behavioral results

Behavioral data revealed that both groups showed similar performance identifying the target words by raising the index finger (p>0.05). Ex-illiterates recognized 58% of the target words, and the controls recognized 62% of these words. On the other hand, the first group made 7% false-recognitions (for non-target words) against 4% false-recognitions among the control subjects. It was decided that all stimuli should be processed because we were not interested in correlating a specific behavioral aspect with a special locus of the brain. The focus was to study which regions were recruited in a similar task by each group of subjects. We can therefore accept that in behaving similarly they were engaged similarly in the task.

### Laterality results

Analysis of the number of sources of activity in the left hemisphere (LH) and the number of sources of activity in the right hemisphere (RH) revealed significant differences between the two groups. (as previously mentioned only sources occurring 150 ms or later following stimulus onset were considered).

In the control group, all the participants had more LH sources than RH sources. Left hemispheric dominance is the expected result in a task with verbal material therefore this is in consonance with previous studies (Simos et al., 1998). On the other hand, in the group of seven ex-illiterates, 3 individuals displayed more LH sources whereas 4 displayed more RH sources (Table 1).

**Table 1:** Comparison of right vs. Left sources of activations between groups.

	Left sources of activation	Right sources of activation
Control group	70.00*±27.531	10.80±8.584
Ex-illiterate group	43.714±23.648**	52.428±23.322**

* Mean total number of left sources and mean total number of right sources in controls differed significantly (p=0.043);

** No significant differences were found between mean total number of left and right sources in ex-illiterates (p>0.05).

Considering the subjects as two groups we found that, in the control group, LH sources represented 86.63% of the activation while RH sources represented only 13.37%. In the ex-illiterate group, LH sources represented 45.47% of the activation and RH sources represented 54.53% of the total number of sources of activation.

Using the previously reported methodology (Breier et al., 2001) the pattern of hemispherical asymmetry was computed and results confirmed that the control group activated significantly more LH sources than RH sources, displaying a significantly asymmetrical pattern (p=0.043) in this recognition task. In the ex-illiterate group however, there was no significant asymmetry between the hemispheres (p>0.05).

It was important to determine the reason for this difference. We compared the number of LH sources between groups and found no differences (p>0.05). In contrast, when comparing the number of RH sources the ex-illiterate group was found to display significantly more RH sources than the control group (p=0.018) (Table 2).These results suggest that the reduction in asymmetry observed was mainly due to an increase in the number of RH sources in the group of ex-illiterate subjects.

**Table 2:** Comparison of sources of activation between groups.

	Control group	Ex-illiterate group
Left sources of activation	70.00±27.531*	43.714±23.648*
Right sources of activation	10.80±8.584	52.428**±23.3227

* No significant differences were found between the mean total number of left sources in controls and ex-illiterates (p>0.05);

** Mean total number of right sources differed significantly between controls and ex-illiterates (p=0.018)

Much of the HAROLD model concerns the pre-frontal cortex. Therefore, we considered the left and right inferior frontal gyrus for analysis, and found that there were only late sources of activation on the right side in the control group, and that they were present both between 150–400 ms and 400–800 ms in the ex-illiterate group (Table 3).

**Table 3:** Comparison of right and left inferior frontal gyrus sources of activation between groups.

	Control group	Ex-illiterate group
Left inferior frontal gyrus sources of activation (150-400 ms)	2.400±3.577*	4.428±7.067*
Left inferior frontal gyrus sources of activation (400-800 ms)	2.000 ±3.464*	3.285±3.773*
Right inferior frontal gyrus sources of activation (150-400 ms)	0.00	2.857**±2.34
Right inferior frontal gyrus sources of activation (400-800 ms)	0.00*	1.285±1.704*

* No significant differences were found between left inferior frontal gyrus sources of activation in controls and ex-illiterates in either time window or in right inferior frontal gyrus in the time window of 400 to 800 ms (p>0.05);

** A significant difference was found between the right inferior frontal gyrus of activation in the time window of 150 to 400 ms (p=0.023).

## Discussion

These results should be interpreted with caution. The groups are small, but we must take into account the difficulties in forming large groups with a similar personal life history of education. We assume that such cases lend stronger evidence then single case reporting and can contribute to the discussion on the three topics stated in the title of this paper.

The critical period can be defined as the best moment for acquiring a certain competence. We have previously reported several differences in brain function and also in brain anatomy that could be attributed to the absence of knowledge of orthography (for general reference see Castro-Caldas, 2004). When subjects decide to learn in adulthood they are confronted with these handicaps. In order to learn and process this new information they recruit areas of the brain that most likely differ from those recruited in childhood. This same group was studied while reading words and the areas involved were different from those used by the controls. However, in this case it was the right parietal lobe that was more active in ex-illiterate subjects (Castro-Caldas et al., 2009). This is similar to findings reported by Newman et al. (2002) for the processing of American Sign Language. Signers that learned sign language before puberty activated the right angular gyrus more than those who learned later. Therefore, our results support the concept of a critical period.

This raises another question: do these results contribute to a better understanding of the concept of cognitive reserve? We believe they do and moreover, they support the argument that we put forward earlier (Castro-Caldas and Guerreiro, 2001). Poorly educated subjects or those from low socioeconomic backgrounds (Raizada et al., 2008) tend to use the brain for information processing in a different way (involving different areas) to those that learned at the regular time. A disease such as Alzheimer’s affects the brain of both groups in a biologically similar way. Nonetheless, the functional result of the lesions can differ between groups because the same areas of the brain are processing different information in each group.

Finally, we should consider the HAROLD model. The notion that recruiting both hemispheres to solve a problem which could potentially be solved by only one side, can be interpreted both as a good strategy or a sign of difficulty in the task. Our task had a component of cued retrieval which can be linked to bilateral frontal activity (Backman et al., 1997). However, this occurred more intensely in the ex-illiterate group and thus our results seem to support the second hypothesis.

Further research is necessary in atypical populations in order to better understand how the typical brain adapts to atypical situations.
